# Supplementary material for: Targeted Delivery of HSP70 to Tumor Cells via Supramolecular Complex Based on HER2-Specific DARPin9_29 and the Barnase:Barstar Pair
Source: Cells. 2024 Feb 9;13(4):317. doi: 10.3390/cells13040317 (PMC10887201; doi:10.3390/cells13040317)
Supplement: Supplementary file 1 [file cells-13-00317-s001.zip › cells-2764802-supplementary.pdf]

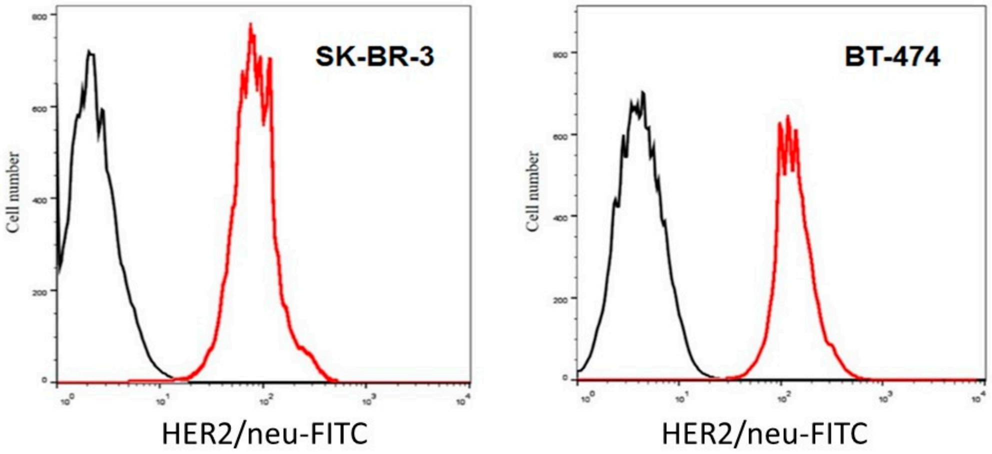

Figure S1. Expression of HER2/neu receptors on the membranes of tumor cell lines SK-BR-3 and BT-474. The cells were stained with DARPin9_29-FITC [6].

Table S1. Averaged fluorescence intensity of HER2/neu receptors on tumor SK-BR-3 and BT474 cell lines stained with DARPin9_29-FITC

**Sample**		**Averaged fluorescence intensity ± standard deviation**
BT-474	autofluorescence	4,2 ±7,7
	HER2/neu	131 ± 72
SK-BR-3	autofluorescence	2,4 ± 3,8
	HER2/neu	67 ± 50

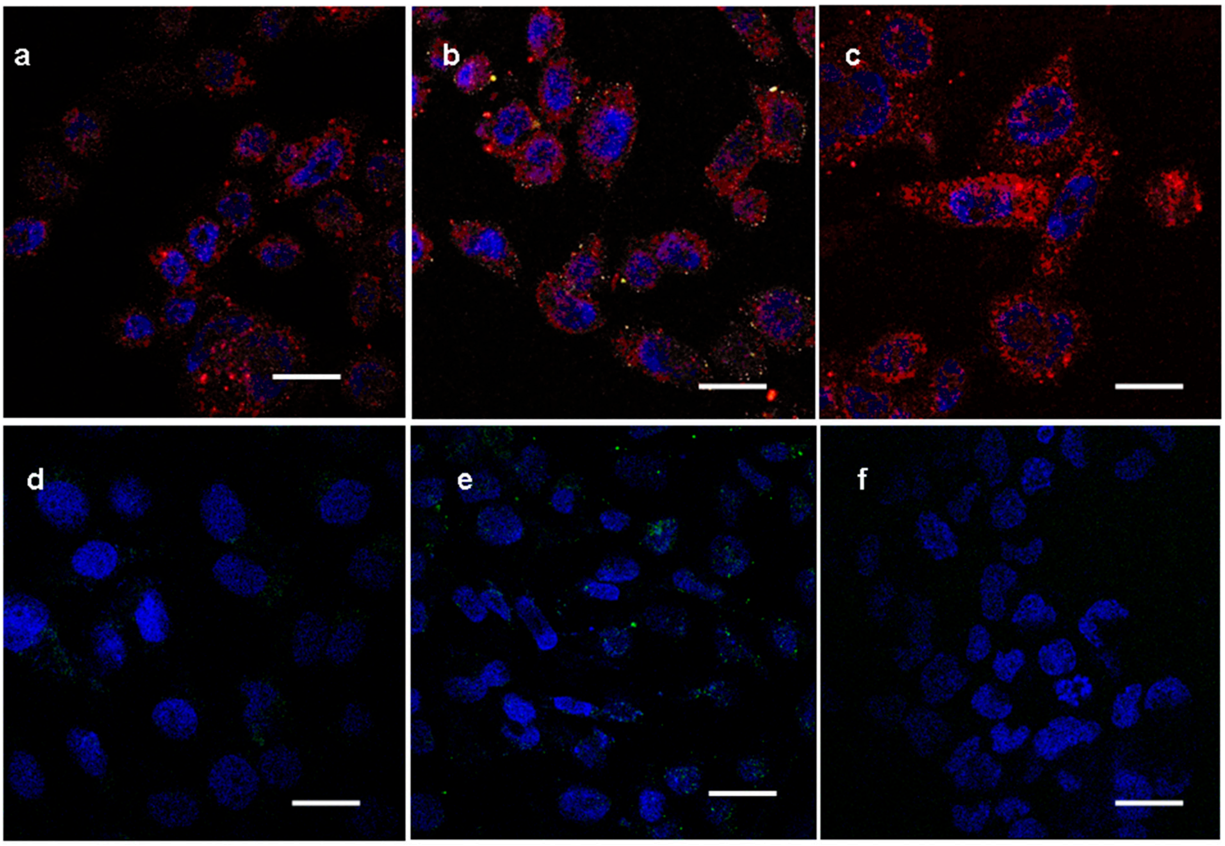

Figure S2. Expression of HSP70 on the surface of SK-BR-3 (a-c) and BT-474 (d-f) cells treated with barstar-HSP70 (a, d) or barstar-HSP70_16 (b, e). The cells treated with barstar-HSP70 (a, d) or barstar-HSP70_16 (unlabeled) were stained with the anti-HSP70 BRM22 antibody (green) and actin (a-c). Cell nuclei were stained with Hoechst 33342 (blue); control cells (c, f) were not treated with barstar-HSP70 or barstar-HSP70_16. Scale bars, 10 μm.

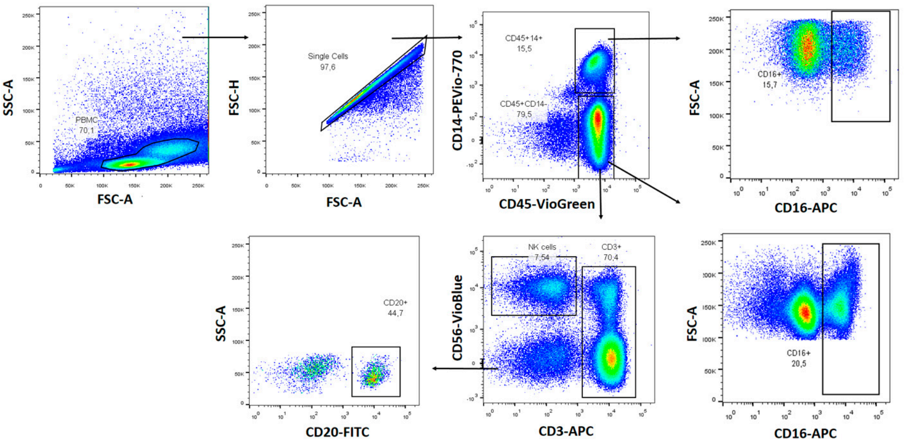

Figure S3. Representative staining of PBMC isolated from a healthy donor. Cells were labeled with CD45, CD14, CD3, CD56, CD16, and CD20 antibodies and analyzed by flow cytometry.
